# Chronic lower extremity wound infection due to *Kerstersia gyiorum* in a patient with Buerger’s disease: a case report

**DOI:** 10.1186/s12879-017-2711-3

**Published:** 2017-09-06

**Authors:** Irmak Baran, Arife Polat Düzgün, İpek Mumcuoğlu, Neriman Aksu

**Affiliations:** 1 Medical Microbiology Department, Ankara Numune Research and Training Hospital, Hacettepe Mahallesi Talatpasa Bulvari No: 44 Altindag, Ankara, Turkey; 2 Clinic of General Surgery, Ankara Numune Research and Training Hospital, Ankara, Turkey; 3 Esat Caddesi 101/3 Kucukesat, 06660, Ankara, Turkey

**Keywords:** *Kerstersia gyiorum*, Buerger’s Disease, Thromboangiitis obliterans, MALDI-TOF MS, 16S rRNA gene sequencing, Chronic wound infection

## Abstract

**Background:**

*Kerstersia gyiorum* is an extremely rare pathogen of human infection. It can cause chronic infection in patients with underlying conditions. It can easily be misdiagnosed if proper diagnostic methods are not used.

**Case presentation:**

A 47-year-old male patient with a history of Buerger’s Disease for 28 years presented to our hospital with an infected chronic wound on foot. The wound was debrided, and the specimen was sent to Microbiology laboratory. Gram staining of the specimen showed abundant polymorphonuclear leukocytes and gram-negative bacilli. Four types of colonies were isolated on blood agar. These were identified as *Kerstersia gyiorum*, *Proteus vulgaris*, *Enterobacter cloacae*, *Morganella morganii* by Maldi Biotyper (Bruker Daltonics, Germany). The identification of *K. gyiorum* was confirmed by 16S ribosomal RNA gene sequencing. The patient was successfully recovered with antimicrobial therapy, surgical debridement, and skin grafting.

**Conclusions:**

This is the first case of wound infection due to *K. gyiorum* in a patient with Buerger’s Disease. We made a brief review of *K. gyiorum* cases up to date. Also, this case is presented to draw attention to the use of new and advanced methods like MALDI-TOF MS and 16S rRNA gene sequencing for identification of rarely isolated species from clinical specimens of patients with chronic infections and with chronic underlying conditions.

## Background

*Kerstersia gyiorum* was first identified in 2003 by Coenye et al. as a distinct species by examination of the isolates obtained from nine clinical specimens such as leg ulcer, sputum, and faeces by cellular fatty acid analysis and 16S rRNA gene sequencing [1]. It belongs to *Alcaligenaceae* family and is closely related to *Alcaligenes*, *Bordetella*, *Achromobacter* spp. [2, 3]. After its first description, there have been publications reporting its isolation from chronic otitis media [2, 4–6], urinary tract infection [3], chronic leg ulcer [2], post-ulcer bacteraemia and sepsis [7] and bronchoalveolar lavage fluid [8].

Here we present a case of chronic foot and ankle infection due to *K. gyiorum* in a 47-year-old patient with Buerger’s Disease and we have made a brief review of *K. gyiorum* cases in literature so far.

## Case presentation

**Fig. 1:**
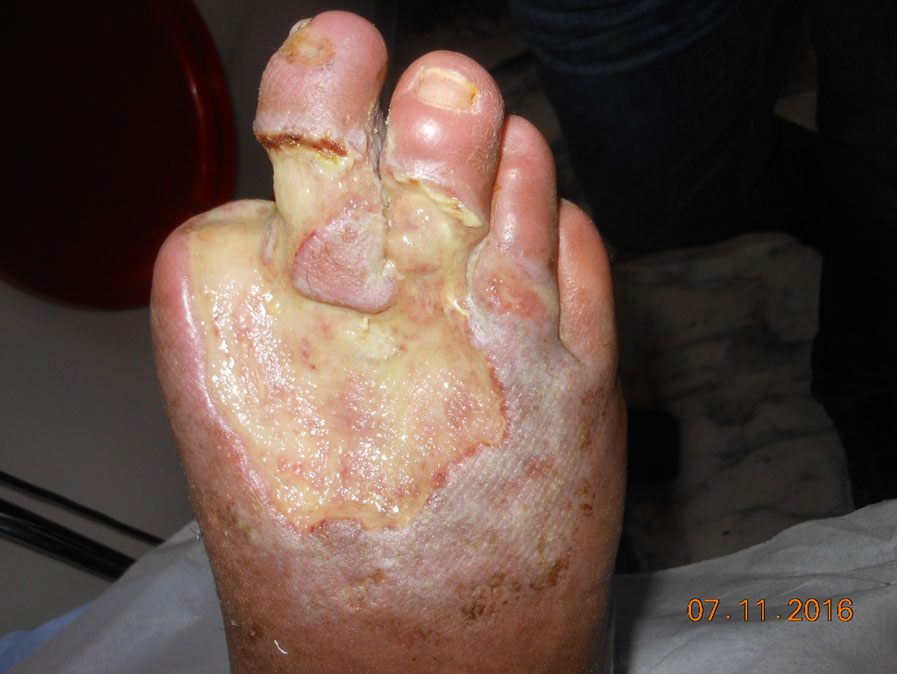
Appearance of wound on the foot

**Fig. 2:**
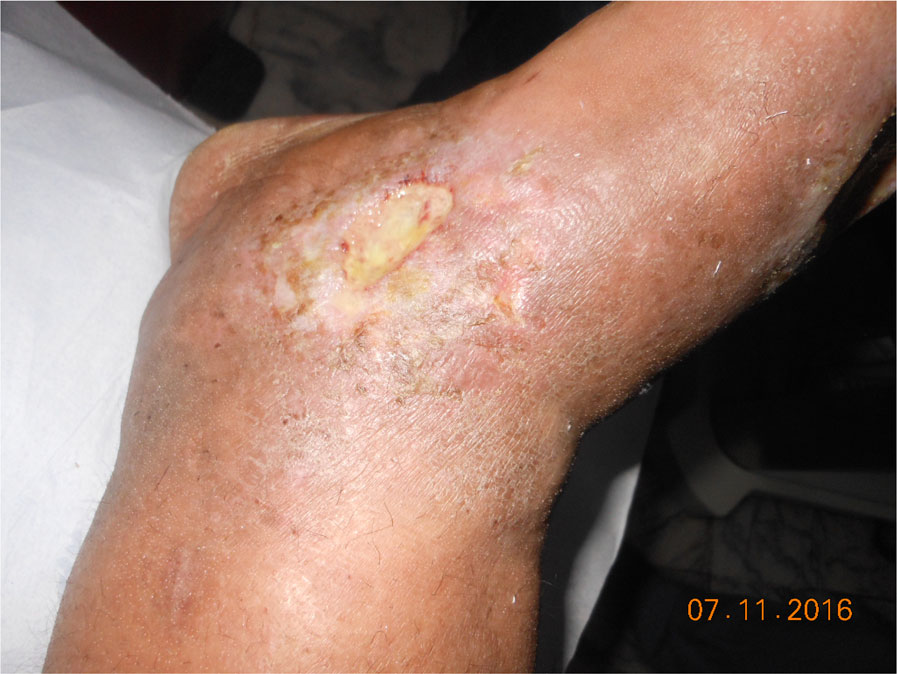
Appearance of wound on ankle

A 47-year-old male patient who was previously followed up at the Chronic Wound Clinic of our hospital presented with a 10 × 15 cm wound on the dorsolateral surface of the right foot and a 2 × 3 cm wound on the outside of the right ankle (Figs. 1 and 2). The patient had a history of Buerger’s Disease (Thromboangiitis obliterans) for 28 years. It has been learned that the patient continues smoking and lives in bad hygienic conditions. The patient received debridement of the wound at the Chronic Wound Clinic, and the sample was sent to our hospital’s Medical Microbiology laboratory for microscopy and culture. Before getting culture results, oral ampicillin sulbactam, ciprofloxacin, and topical mupirocin treatment were started to the patient empirically.

When we questioned the patient he said that he had previously applied to the Chronic Wound Clinic nine months before this admission. The patient had first come to the Department of Cardiovascular Surgery because stem cell treatment was being considered. The patient had his toe amputated at another medical centre one month before that. The patient was referred to the Chronic Wound Clinic for the treatment of the infected chronic wound at his foot before stem cell treatment. In the blood tests performed at the first visit, the white blood cell count was 9500/μL (65.1% neutrophils), hemoglobin 12.3 g/dl, hematocrit 38.9%, platelets 348.000/μL. Both the patient’s fasting blood glucose (95 mg/dL) and % HbA1c (6.1%) levels were found to be normal. The patient had higher than normal levels of CRP (12 mg/L) and erythrocyte sedimentation rate (55 mm/h). The patient’s coagulation tests were within normal limits (aPTT 31.8 s, prothrombin time 11 s, INR 0.99). The patient’s HBsAg, Anti-HIV, and Anti-HCV tests were shown to be negative with ELISA method (ETI-MAX 3000 analyzer; DiaSorin S.p.A., UK). The angiography of the leg showed left deep femoral artery had thin calibration, the right superficial femoral artery was occluded at the level of the ½ middle of the thigh, and there were intense collateral arteries on leg and thigh. Microscopic examination of the specimen taken from the wound revealed 4–5 polymorphonuclear leucocytes in each area. *Pseudomonas aeruginosa* was isolated from wound culture. When antibiotic sensitivity tests were made this microorganism was found to be sensitive to tobramycin, colistin, ceftazidime, and gentamicin. The patient was given iv ceftazidime and metronidazole treatment for 14 days. The patient was hospitalized for one month, and treated with hyperbaric oxygen therapy. After skin grafting, the patient was discharged.

This time the previous graft was found to be lysed, and the wound was re-infected. The blood tests were performed and white blood cells were found 8200/μL (70% neutrophils), hemoglobin 14 g/dl, hematocrit 43.7%, and platelets 306.000 /μL. The patient’s CRP (7 mg / L) and erythrocyte sedimentation rate (65 mm / h) were found to be high.

Plenty of polymorphonuclear leukocytes and gram-negative bacilli were present in the Gram stained sample taken from the patient’s wound which was sent to our hospital’s Medical Microbiology laboratory. The material was inoculated on to blood agar and Eosin methylene blue (EMB) agar and incubated. Four different types of colonies were identified after 24 h of incubation at 37 °C on bloody agar and EMB agar. Out of these four colony types, the colonies predominantly grown on blood agar were light gray, had a dry appearance, and were large colonies with indented edges that tended to merge with each other, resembling *Alcaligenes*. However, unlike *Alcaligenes* there was no fruity odour and oxidase test was negative. These colonies were found to be indole-negative, urease-negative and strongly catalase-positive. The colonies grown on blood agar with second density were gray coloured and had swarming around the colony, were indole positive, urease positive, oxidase negative, hydrogen sulphide producing colonies. The third colony type was dirty-white-coloured, mucoid with smooth edges. They were indole negative, Voges-Proskauer test positive. The fourth colony type was smooth-edged, whitish mucoid colonies. Urease test was positive, indole positive and Voges-Proskauer test was negative. On EMB medium a mixed growth of one lactose-positive and three lactose-negative colonies corresponding to these four types of colonies was observed. Single colony passages were made from each of these four colony types in order to identify with Maldi Biotyper (Bruker Daltonics, Germany) system, a matrix-assisted laser desorption ionization-time of flight mass spectrometry (MALDI-TOF MS) system in our Medical Microbiology Laboratory. Antibiotic susceptibility tests of the bacteria were carried out on a Phoenix 100 (Beckton Dickinson, USA) device.

**Fig. 3:**
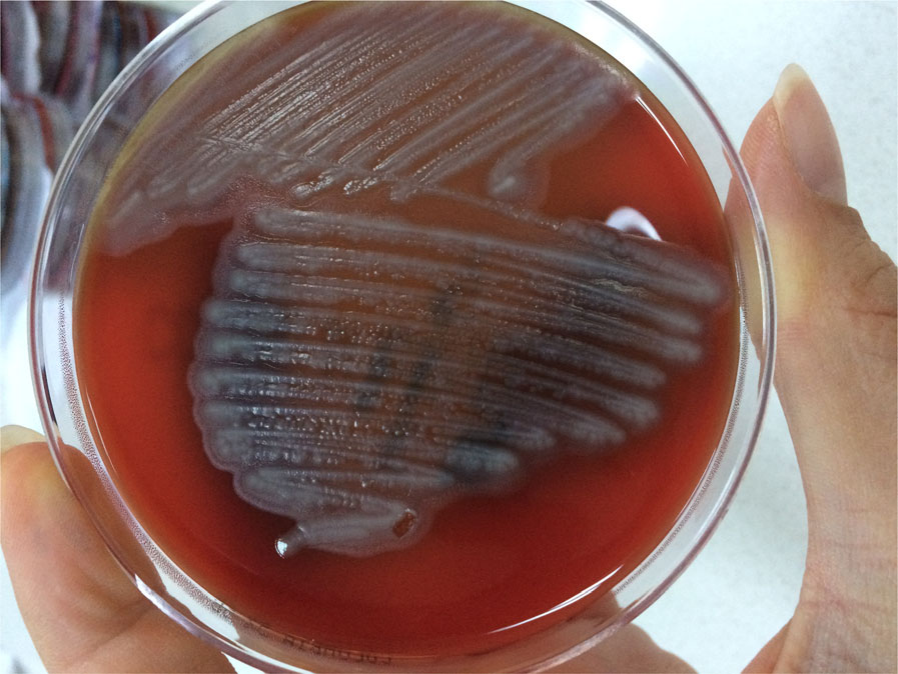
*Kerstersia gyiorum* on blood agar

**Fig. 4:**
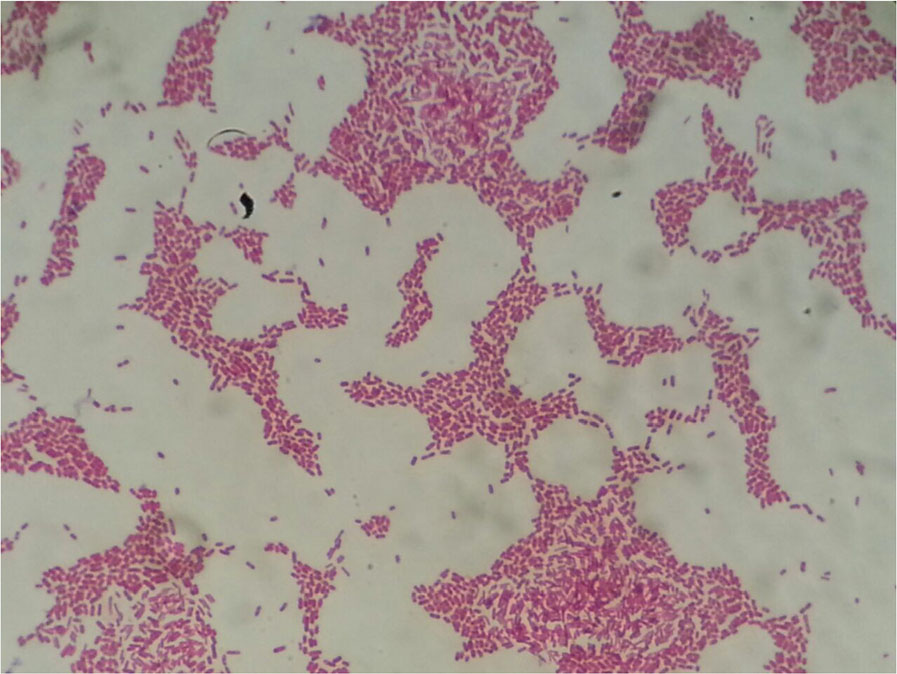
Gram stain from the pure culture of *Kerstersia gyiorum*. Gram-negative, short rods were seen

The passages made from the first colony type produced dry, light gray, opaque colonies, with irregularly indented, protruding edges, and swarming around the colonies on blood agar (Fig. 3), and lactose-negative colonies on EMB. Gram-negative, short bacilli were seen in Gram staining of colonies from blood agar (Fig. 4). These colonies were identified as *Kerstersia gyiorum* by Maldi Biotyper (Bruker Daltonics, Germany) system with 2.348 Biotyper score (excellent identification). The identification of *K. gyiorum* with Maldi Biotyper was confirmed by 16S ribosomal RNA gene sequencing according to the predefined methodology [9, 10]. Briefly, the following steps were taken for this procedure. DNA was isolated from pure culture using DNeasy Blood & tissue kit (Qiagen, Germany). 16S rRNA gene amplification was performed using universal 27F (AGAGTTTGATCMTGGCTCAG) and 1492R (GGTTACCTTGTTACGACTT) primers. In the PCR reaction, 1XTaq buffer, 2 mM MgCl 2, 0.2 mM dNTP, 0.4 pmol of primers and 1.25 U Taq polymerase (Thermo Fisher Scientific, USA) were used in a volume of 50 μL. DNA sequencing of the resulting product was performed using the Bigdye Cycle Sequencing Kit v.3.1 (Applied Biosystems, USA). Sequence analyzes were compared to the GenBank NCBI gene library data using the BLAST program (https://blast.ncbi.nlm.nih.gov/Blast.cgi). The sequence was found 100% identical to *K. gyiorum*. Antibiotic susceptibility tests for *K. gyiorum* were performed using the Phoenix 100 (Beckton Dickinson, USA) device and the E-test (Liofilchem, Italy) for six antimicrobial agents (ceftriaxone, ciprofloxacin, colistin, gentamicin, imipenem, trimethoprim-sulfamethoxazole) on Mueller-Hinton agar in accordance with the manufacturer’s specifications. The results were evaluated according to the MIC breakpoints established by Clinical and Laboratory Standards Institute for other non-*Enterobacteriaceae* [11]. The results of antibiotic susceptibility tests for *K. gyiorum* are shown in Table 1.

**Table 1:** Antimicrobial susceptibility profile of *Kerstersia gyiorum* as determined by Phoenix 100 and E-test

	Phoenix 100		E-test	
Antimicrobial agent	MIC (μg/mL)	Result	MIC (μg/mL)	Result
Amikacin	≤4	Susceptible	–	–
Aztreonam	≤1	Susceptible	–	–
Ceftazidime	≤0.5	Susceptible	–	–
Ceftriaxone	–	–	0.25	Susceptible
Ciprofloxacin	≤0.125	Susceptible	0.25	Susceptible
Colistin	>4	Resistant	>256	Resistant
Gentamicin	2	Susceptible	1	Susceptible
Imipenem	–	–	2	Susceptible
Meropenem	≤0.125	Susceptible	–	–
Netilmicin	2	Susceptible	–	–
Piperacillin	≤4	Susceptible	–	–
Piperacillin-tazobactam	≤4/4	Susceptible	–	–
Trimethoprim-sulfamethoxazole	≤1/19	Susceptible	≤2/38	Susceptible

Other microorganisms were identified as *Proteus vulgaris*, *Enterobacter cloacae*, *Morganella morganii* by Maldi Biotyper system, respectively. Antibiotic susceptibility results of *P. vulgaris*, *E. cloacae*, *M. morganii* are shown in Table 2.

**Table 2:** Antimicrobial susceptibility results of *Proteus vulgaris*, *Enterobacter cloacae*, *Morganella morganii*

	*Proteus vulgaris*	*Enterobacter cloacae*	*Morganella morganii*
Amikacin	Susceptible	Susceptible	Susceptible
Amoxicillin-clavulanic acid	Susceptible	Resistant	Resistant
Ampicillin	Resistant	Resistant	Resistant
Ampicillin-sulbactam	Susceptible	Susceptible	Susceptible
Ertapenem	Susceptible	Susceptible	Susceptible
Gentamicin	Susceptible	Susceptible	Susceptible
Imipenem	Intermediate	Susceptible	Susceptible
Levofloxacin	Susceptible	Susceptible	Susceptible
Meropenem	Susceptible	Susceptible	Susceptible
Piperacillin-tazobactam	Susceptible	Susceptible	Susceptible
Cefepime	Susceptible	Susceptible	Susceptible
Cefoxitin	Susceptible	Susceptible	Susceptible
Cefotaxime	Susceptible	Susceptible	Susceptible
Ceftriaxone	Resistant	Susceptible	Susceptible
Cefuroxime	Susceptible	Susceptible	Susceptible
Ciprofloxacin	Susceptible	Susceptible	Susceptible
Tobramycin	Susceptible	Susceptible	Susceptible
Trimethoprim-sulfamethoxazole	Resistant	Resistant	Susceptible

**Fig. 5:**
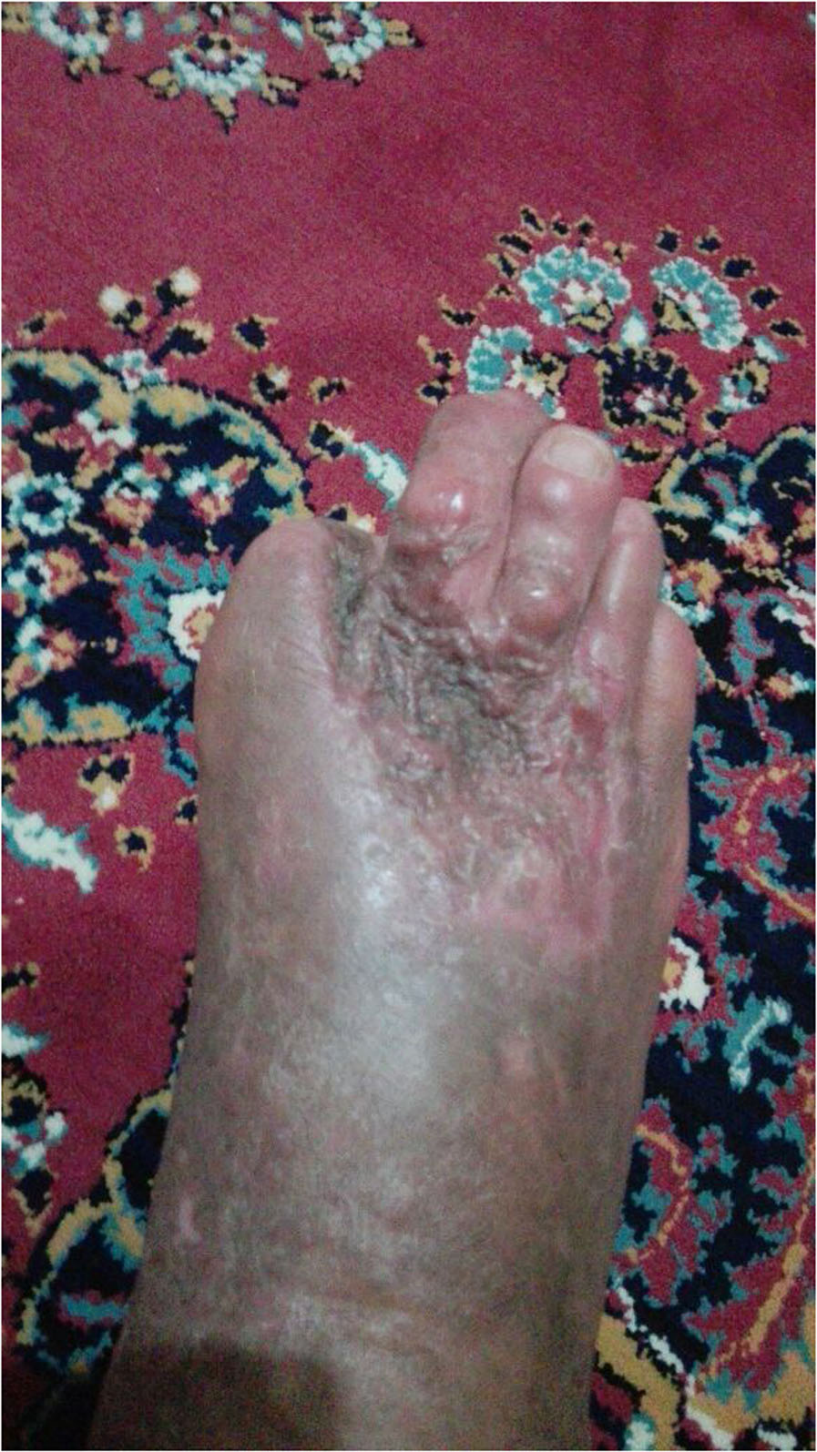
Appearance of the patient’s foot at the three-month follow-up

**Fig. 6:**
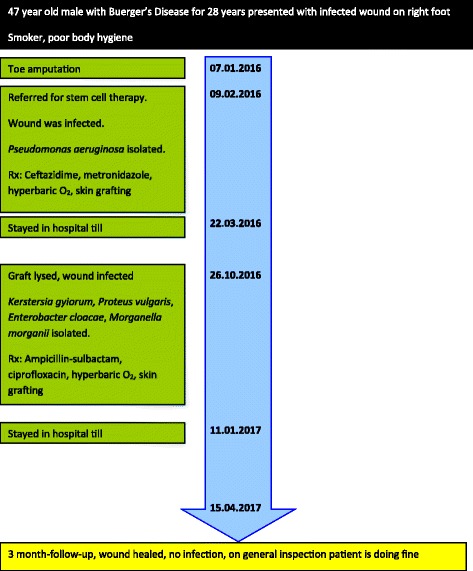
A timeline of all events since patient’s first hospitalization

Oral ampicillin-sulbactam and ciprofloxacin treatment was continued because the microorganisms were susceptible to these antibiotics. Hyperbaric oxygen therapy was used. Wound debridement and skin grafting were applied. The patient was discharged after one month of hospitalization. The patient was doing well in follow-up examination three-months later (Fig. 5). Figure 6 shows a timeline of events.

## Discussion

*K. gyiorum*’s name was derived from the Greek word “gyion”, which means “limb” since it was often isolated from leg and ankle wounds when it was first described [1–3, 6, 7]. *K. gyiorum* belongs to *Alcaligenaceae* family, and it is related to *Alcaligenes*, *Bordatella, Achromobacter,* and *Pigmentiphaga* generas [1, 3–7]. *K. gyiorum* colonies are known to show an appearance similar to *Alcaligenes faecalis.* However, *K. gyiorum* isolates are oxidase negative and lack the characteristic fruity odor [3, 7]. *K. gyiorum* is highly catalase positive but urease and β-galactosidase negative [1].

When we conducted a search for “*Kerstersia gyiorum* case report” on PubMed and Medline we found that there were 9 cases since the first time it was defined in 2003 [2–8] Table 3 shows clinical features of these case reports. In 2012, Vandamme et al. identified *Kerstersia similis*, a close species, from the neck abscess of a 54-year-old patient [12].

**Table 3:** Clinical features of reported cases of *Kerstersia* spp. after first identification in 2003 by Coenye et al. [1] (Source: PubMed, Medline)

Year of publication	Reference number	Authors	Patient age	Gender	Smoking history	Clinical condition	Source	Polymicrobial/monomicrobial	Isolated Species	Antibiotic treatment	Outcome
2012	4	Almuzara et al.	16	Male	Absent	Complicated chronic otitis media	Bezold’s abscess	Monomicrobial	*Kerstersia gyiorum*	Iv ampicillin-sulbactam and ceftriaxone (3 days) Oral ciprofloxacin and amoxicillin-clavulanic acid	Recovered
2013	2	Pence et al.	55	Male	Present	Chronic otitis medi	Mastoid cavity specimen	Polymicrobial	*Kerstersia gyiorum* *Corynebacterium amycolatum*	Trimethoprim-sulfamethoxazole (14 days)	Recovered
2013	2	Pence et al.	61	Female	Absent	Lower leg ulcer	Wound specimen swab	Polymicrobial	*Kerstersia gyiorum* *Morganella morganii*	Ciprofloxacin (10 days)	Unknown
2014	8	Deutscher et al.	63	Female	Absent-	Ventilator-dependent chronic respiratory failure, Chronic tracheostomy	Bronchoalveolar lavage	Polymicrobial	*Kerstersia gyiorum,* *Pseudomonas aeruginosa, Stenotrophomonas maltophilia*	Piperacillin-tazobactam, doripenem, ciprofloxacin, ceftazidime, colistin	Died from complications
2014	6	Mwalutende et al.	53	Male	Present	Chronic suppurative otitis media	Ear swab	Polymicrobial	*Kerstersia gyiorum,* *Proteus mirabilis*	Ciprofloxacin ear drops	Recovered
2014	6	Mwalutende et al.	33	Male	Absent	Chronic suppurative otitis media	Ear swab	Polymicrobial	*Kerstersia gyiorum,* *Escherichia coli* *Staphylococcus aureus*	Ciprofloxacin ear drops	Recovered
2015	5	Uysal et al.	25	Male	Absent	Chronic suppurative otitis media	Ear swab	Polymicrobial	*Kerstersia gyiorum* *Pseudomonas aeruginosa,*	Imipenem (10 days)	Recovered
2015	7	Bostwick et al.	69	Female	Absent	Chronic lower extremity ulcer, bacteremia, sepsis	Blood culture	Monomicrobial	*Kerstersia gyiorum*	Ciprofloxacin, clindamycin (14 days)	Recovered
2016	3	Ogawa et al.	82	Male	Absent	Urinary tract infection	Urine	Polymicrobial	*Kerstersia gyiorum* *Proteus vulgaris*	Levofloxacin (5 days)	Recovered
2012	12	Vandamme et al.	54	Male	Unknown	Unknown	Neck abscess	Unknown	*Kerstersia similis*	Unknown	Unknown

In our case *K. gyiorum* grew with other types of microorganisms from wound specimen. This was also observed by previous researchers who have reported cases of *K. gyiorum* [2–8]. In seven of the nine cases that have been reported so far there were polymicrobial infections. *K. gyiorum* was isolated from urinary tract infection together with *P. vulgaris* [3], from chronic otitis media with *Corynebacterium amycolatum*, from chronic lower extremity wound with *Morganella spp.* [2], from bronchoalveolar lavage fluid with *Pseudomonas aeruginosa* and *Stenotrophomonas maltophilia* [8], from chronic suppurative otitis media with *P. aeruginosa* in a case report [5] from our country, and in two different cases from chronic suppurative otitis media with *Proteus mirabilis* and from chronic suppurative otitis media with *Staphylococcus aureus* and *Escherichia coli* [6]. In our case, *K. gyiorum* was seen to be grown more dominantly than other microorganisms in the culture plate. However, since there are a very limited number of reports in the literature on this microorganism, it is difficult to predict the effect of *K. gyiorum* in the disease process in a polymicrobial infection [2]. The virulence factors of this microorganism should be investigated [6]. In our case, the patient’s condition got better after antimicrobial therapy. Ogawa et al. reported that *K. gyiorum* is prone to cause infection in situations predisposing to polymicrobial infection because *Achromobacter* and *Alcaligenes spp*. which are closely related have the same tendency [3].

If we look at the results of antibiotic susceptibility, our *K. gyiorum* isolate was found to be susceptible to aminoglycosides, ciprofloxacin, imipenem and meropenem and broad spectrum cephalosporins. These results were similar to Coenye et al. [1] and Almuzara et al.’s results [4]. However, Pence et al. [2], Uysal et al. [5], Mwalutende et al. [6], Bostwick et al. [7], Deutscher et al. [8], found resistance to ciprofloxacin in *K. gyiorum* isolates.

Our patient was a smoker. He had Buerger’s Disease (Thromboangiitis obliterans) for 28 years due to smoking. Thromboangiitis obliterans is a non-atherosclerotic inflammatory disease affecting the small and medium vessels in the limbs and causes circulatory problems [13]. In this disease, ulcers that do not heal especially at the distal end of the extremities are seen [13, 14]. The chronic suppurative otitis media case due to *K. gyiorum* that Mwalutende et al. reported was a chronic smoker [6]. Pence et al. also reported chronic cigarette smoking in their case [2]. We believe that there may be a relationship between chronic smoking and infection due to *K. gyiorum*. However, further work is needed in order to test this hypothesis.

Previous researchers who reported case reports of *K. gyiorum* reported that the associated infection usually develops on a long-standing inflammatory condition [2–4, 6, 8]. Our patient had long-term lower extremity ulcers due to Buerger’s Disease.

In our case, we observed that on blood agar *K. gyiorum* forms colonies with a irregular spreading edge morphology. Pence et al., Deutscher et al., and Bostwick et al. reported that they observed colonies with similar appearance to ours [2, 7, 8]. We think that these phenotypical features may be typical for *K. gyiorum*. This morphology can be used to distinguish *K. gyiorum* from *Acinetobacter* spp., which is also a non-fermentative and oxidase-negative bacterium as suggested by previous researchers [2, 8]. Pence et al. referred to the formation of lavender pigment on MacConkey agar [2]. We did not see such pigment formation in our own case. However we used EMB instead of MacConkey agar. In our opinion, the appearance of such a colony type after inoculation of a specimen from a site of chronic inflammation should suggest *K. gyiorum* infection.

Introduction of molecular and genetic identification methods to clinical microbiology has increased the detection of new and rare bacteria in clinical specimens. MALDI-TOF MS and 16S rRNA gene sequencing are two of these new and advanced methods [2, 6, 8]. In our case we detected the presence of *K. gyiorum* by using MALDI-TOF MS. The reliance on solely biochemical identification methods or automated identification systems may lead to misidentification of *K. gyiorum*, because it has common biochemical features with more commonly detected pathogens such as *Acinetobacter* spp. [5–7]. Also *K. gyiorum* is phenotypically similar to *Alcaligenes faecalis* and it may go unnoticed if proper identification methods are not used [5]. The increase in the identification of new and previously unrecognized bacteria has led to two important situations in clinical microbiology laboratories. The first is to determine whether these microorganisms are pathogens or contaminants, and the second is to correctly determine the antibiotic susceptibilities of these microorganisms [2]. The co-operation of the clinician and the laboratory is required to achieve these. As the data in the literature will increase with the studies to be done on this subject, a more accurate perspective will be obtained for approaching these cases. Besides, it is difficult to identify such rare microorganisms by conventional methods. Therefore, when the pathogen cannot be identified by routine biochemical methods in cases of chronic infections that do not heal, MALDI-TOF MS and 16S rRNA gene sequencing are emerging as fast and reliable alternatives [5, 8].

## Conclusions

We think that *K. gyiorum* should be kept in mind as a possible agent associated with chronic infected lower extremity ulcers in patients with Buerger’s Disease.

## Abbreviations

16S rRNA: 16S ribosomal ribonucleic acid
Anti-HCV: Anti hepatitis C virus antibodies
Anti-HIV: Anti human immunodeficiency virus antibodies
aPTT: Activated partial thromboplastin time
CRP: C-reactive protein
ELISA: Enzyme-linked immunosorbent assay
EMB: Eosin methylene blue
E-test: Epsilometer test
HbA1c: Glycated haemoglobin
HBsAg: Surface antigen of the hepatitis B virus
INR: International normalized ratio
MALDI-TOF MS: Matrix-assisted laser desorption ionization-time of flight mass spectrometry
MIC: Minimum inhibitory concentration
NCBI: National Center for Biotechnology Information
PCR: Polymerase chain reaction
